# Surfactin inducing mitochondria-dependent ROS to activate MAPKs, NF-κB and inflammasomes in macrophages for adjuvant activity

**DOI:** 10.1038/srep39303

**Published:** 2016-12-14

**Authors:** Ping Gan, Zhenqiu Gao, Xiuyun Zhao, Gaofu Qi

**Affiliations:** 1 College of Life Science and Technology, Huazhong Agricultural University, Wuhan 430070, China; 2 Guilin Medical University, No. 1 Zhiyuan Road, Lingui District, Guilin, China; 3 School of Pharmacy, Yancheng Teachers’ University, Xiwang Road, Yancheng, 224051, China; 4 Biomedical Center, Huazhong Agricultural University, Wuhan 430070, China

## Abstract

Surfactin, a natural lipopeptide, can be used both as parenteral and non-parenteral adjuvant for eliciting immune response. However, the mechanisms that confer its adjuvant properties have not been fully explored. By staining with NHS-Rhodamine B labeled surfactin and Mito-Tracker Green, we found surfactin could penetrate into macrophages to bind with mitochondria, following induce ROS that could be inhibited by mitochondria-dependent ROS inhibitor. ROS enhanced p38 MAPK and JNK expression, as well their phorsphorylation, following activated NF-κB nuclear translocation in macrophages that was obviously inhibited by mitochondria-dependent ROS inhibitor. However, inhibition of ROS production only weakened p38 MAPK and JNK expression, but not their phosphorylation in macrophages. As a result, surfaction could activate NF-κB to release TNF-α by the mitochondria-dependent ROS signalling pathway. ROS also induced macrophages apoptosis to release endogenous danger signals, following activated inflammasomes of NLRP1, NLRP3, IPAF and AIM2 *in vitro* and only NLRP1 *in vivo*, as well caspase-1 and IL-1 in macrophages, which were significantly inhibited by pre-treatment with ROS inhibitors. Collectively, surfactin as a kind of non-pathogen-associated molecular patterns, modulates host innate immunity by multiple signalling pathways, including induction of mitochondria-dependent ROS, activating MAPKs and NF-κB, and inducing cell apoptosis to realease endogenous danger signals for activation of inflammasomes.

New adjuvants for vaccination is very important for successfully fighting against many life-threatening infectious diseases and cancers. Subunit vaccines (*e.g.*, protein and peptide vaccines) can increase vaccine safety because they purposely exclude possible allergens, toxins, and virulent molecular domains of a pathogen. However, the subunit antigen alone is frequently insufficient to elicit a protective immune response. Thereby, adjuvants are very critical for use with subunit vaccines to increase the magnitude and duration of adaptive immunity^1^.

Many immunostimulatory adjuvants (*e.g.* endotoxin, peptidoglycan or unmethylated CpG motifs) are pathogen-associated molecular patterns (PAMPs) or their synthetic analogues, which trigger pattern recognition receptors (PRRs) of the innate immune system such as Toll-like receptors, following activate mitogen activated protein kinases (MAPKs) and transcription factor NF-κB^2^,^3^. Some other adjuvants like alum, can activate inflammasomes through damaging tissue to release damage-associated molecular patterns (DAMPs) like uric acid, ATP or host DNA. DAMPs can be sensed by intracellular PRRs such as NOD-like receptors, following upregulate expression of various pro-inflammatory factors including IL-1β^2^. IL-1β is a potent inflammatory cytokine that can stimulate Th2-cell proliferation and boost antibody production^4^.

Inflammasomes activate the protease caspase-1, which can cleave pro-IL-1 into bioactive IL-1β. Several types of inflammasomes (*e.g.* NLRP1, NLRP3, IPAF and AIM2) have been described, each containing a specific danger sensor that mediates recognition of a distinct stimulus or a set of stimuli, including ATP, monosodium urate crystals, the adjuvant alum, as well as various bacterial products^4^,^5^. One event required for inflammasome activation is the generation of reactive oxygen species (ROS), as most known inflammasome stimuli trigger ROS generation and treatment with various ROS scavengers blocks inflammasome activation in response to the agonists^2^,^6^.

We recently showed that Surfactin (SFN), a natural lipopeptide produced by *Bacillus*, can be used both as parenteral and non-parenteral adjuvant for various protein or peptide antigens^7^,^8^. SFN is a kind of non-pathogen-associated molecular patterns that helps to elicit a balanced Th1/Th2 response *in vivo*^7^. Notably, the mucosal adjuvant activity of SFN is comparable to cholera toxin, the “gold standard” of mucosal adjuvanticity^8^. After treatment with SFN, a robust of ROS was found in dendritic cells and macrophages, accompanying with increased expression of MHCI/II molecules and secretion of Th1/Th2 cytokines (TNF-α and IL-10)^7^,^8^. Interestingly, SFN also triggers a robust of ROS in plant or fungal cells. SFN induces ROS to elicit plant systemic resistance, possible by activation of the intracellular NOD receptors of plant cells^9^. As well, SFN stimulates ROS to induce cells apoptosis in fungus^10^. Moreover, SFN can penetrate into macrophages^7^, thereby it is possible to be recognized by intracellular NOD-like receptors to activate inflammasomes. However, the mechanisms that confer these immunomodulatory properties to SFN has not been fully explored.

Using immunization studies *in vitro* and *in vivo*, we demonstrate in this paper that SFN, a kind of non-pathogen-associated molecular patterns, acts as an agonist inducing mitochondria-dependent ROS to activate MAPKs, NF-κB and inflammasomes in macrophages. This study will provide an insight into the mode of surfactin action to trigger immune response.

## Results

### Surfactin inducing ROS in macrophages *in vitro* and *in vivo*

**Figure 1:**
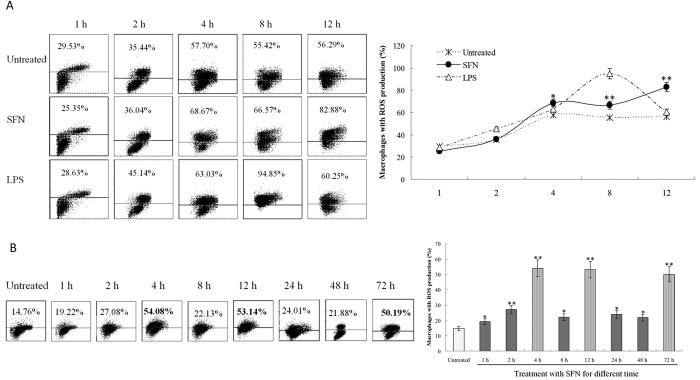
Surfactin inducing ROS in macrophages. (**A**) ROS produced by Raw 264.7 cells *in vitro*. After stimulation with SFN (5 μg/ml), ROS were obviously induced in macrophages that are similar to LPS control. (**B**) ROS produced by mouse peritoneal macrophages *in vivo*. After stimulation with SFN (100 μg/mouse), ROS repeated with increase and wane in the mouse peritoneal macrophages. Double stars (p < 0.01) and single star (p < 0.05) mean the significant difference between SFN treatment and untreated.

After stimulation with surfactin (SFN), ROS were determined by staining with DCFH-DA and analyzed by FACS. *In vitro* stimulation with SFN (5 μg/ml) more than 4 h, ROS were obviously induced in Raw 264.7 macrophages that are similar to the positive control of lipopolysaccharide (LPS) (Fig. 1A). There was also an increase (from 29.53% to 57.70%) of ROS production in untreated cells up to 4 h. This is due to the characteristics of macrophages, which are easy to produce ROS as phagocytes. Thereby, macrophages without treatment by SFN also produced ROS, this might be influenced by the harmful metabolic substances in the culture; however, the ROS production was significantly higher in the SFN-treated group than the untreated control.

*In vivo* stimulation with SFN (100 μg/mouse) could rapidly induce ROS in the peritoneal marophages of mice post-stimulation for 4 h, and approximately waned to the level similar to the untreated group post-stimulation for 8 h (Fig. 1B). After stimulation for 12 h, ROS significantly increased again, then decreased to the level similar to the untreated group at 24 h and 48 h, following significantly increased again at 72 h (Fig. 1B). After ROS is induced, the peritoneal macrophages will produce anti-oxidant substances such as GSH and vitamin C, and anti-oxidant enzymes such as SOD to clear excessive ROS or go into the procedure of apoptosis as a feedback to the high level of cellular ROS. However, the residual SFN might consistently stimulate peritoneal macrophages to produce ROS, with a high content at the time point of 4, 12 and 72 h, respectively. The results suggest that SFN can induce ROS in macrophages both *in vitro* and *in vivo*. In the following studies, we selected the time points of 48 h (low ROS production) and 72 h (high ROS production) for *in vivo* assay.

### Surfactin inducing ROS via mitochondria

**Figure 2:**
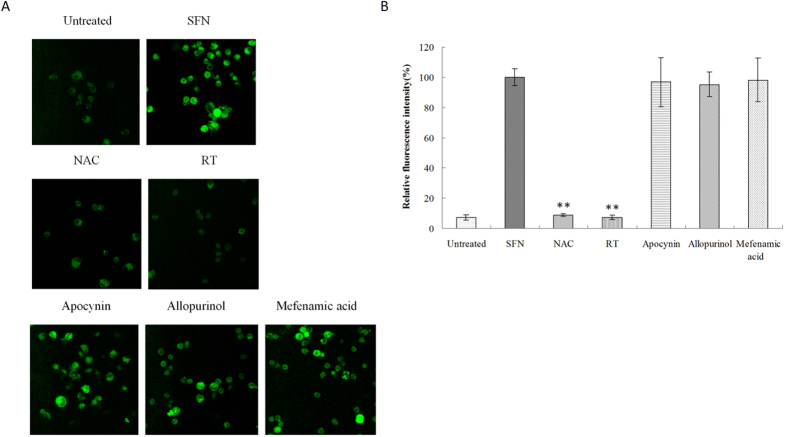
ROS in macrophages pre-treated with different ROS inhibitors. Raw 264.7 cells were pre-incubated with different ROS inhibitors, then stimulated with SFN. (**A**) ROS were determined by staining with DCFH-DA and observed by Confocal microscopy (Magnification is 40X). (**B**) The relative fluorescence intensity was quantified, and it was found only NAC or RT could significantly inhibit ROS production in macrophages. Double stars (p < 0.01) mean the significant difference between pre-treatment with ROS inhibitors and SFN.

Raw 264.7 cells were incubated with different ROS inhibitors, then stimulated with SFN (5 μg/ml). After staining with DCFH-DA, the results showed both NAC (universal ROS inhibitor) and Rotenone plus TTFA (RT, mitochondria-dependent ROS inhibitor) could significantly inhibit SFN-induced ROS in macrophages (Fig. 2). Neither apocynin (NADPH-dependent ROS inhibitor) nor allopurinol (Xanthine oxidase-dependent ROS inhibitor) and mefenamic acid (Cyclooxygenase-dependent ROS inhibitor), could obviously inhibit SFN-induced ROS production in macrophages when compared to SFN treatment (Fig. 2). These results indicate that SFN induces ROS via mitochondria in macrophages.

### Surfactin binding to mitochondria

**Figure 3:**
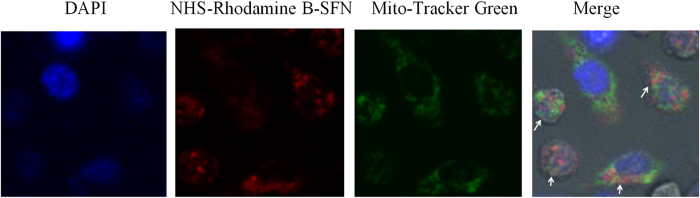
Surfactin co-located with mitochondria in macrophages. Raw 264.7 cells were incubated with NHS-Rhodamine B-labeled SFN and Mito-Tracker, then observed by Confocal microscopy (Magnification is 63×). NHS-Rhodamine B-labeled SFN (red fluorescence) co-located with the mitochondria-specific dye of Mito-Tracker (green fluorescence) in the cytoplasm of macrophages (directed by white arrows).

Our previous studies showed that SFN could penetrate into macrophages^7^, and bind to the mitochondria of fungal cells^10^. Thereby, we deduced that SFN could also bind to mitochondria to induce ROS in macrophages. After observed by Confocal microscopy, we found NHS-Rhodamine B-labeled SFN (red fluorescence) co-located with mitochondria-specific dye of Mito-Tracker (green fluorescence) in the cytoplasm of macrophages (Fig. 3). The results clearly suggest that SFN penetrates into cells and binds to mitochondria to induce ROS in macrophages.

### Surfactin activating MAPKs signalling pathway in macrophages

**Figure 4:**
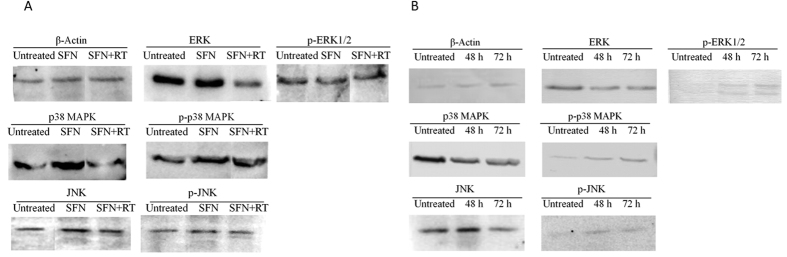
Western blotting for analysis of MAPKs in macrophages stimulated with surfactin *in vitro* and *in vivo*. (**A**) Stimulation with SFN *in vitro*. SFN (5 μg/ml) obviously increased p38 MAPK and p-p38 MAPK, and slightly increase JNK and p-JNK in macrophages when compared to untreated control. ROS inhibitor could decrease p38 MAPK and JNK expression but not their phosphorylation in macrophages. (**B**) Stimulation with SFN *in vivo*. SFN (100 μg/mouse) promoted phosphorylation of ERK, p38 MAPK and JNK in the mouse peritoneal macrophages.

ROS can activate MAPKs family including extracellular signal-regulated kinase (ERK), p38 mitogen-activated protein kinase (p38 MAPK) and Jun N-terminal kinase (c-JNK) that regulate inflammation by activation of NF-κB^11^,^12^. Analyzed by Western bloting, we found stimulation with SFN (5 μg/ml) *in vitro* could not increase either ERK nor p-ERK1/2 in macrophages when compared to the untreated control (Fig. 4A). On the other hand, SNF could obviously increase both p38 MAPK and p-p38 MAPK, and slightly increase both JNK and p-JNK in macrophages (Fig. 4A). Inhibition of ROS by RT could decrease the expression both of p38 MAPK and JNK but not their phosphorylation in macrophages when compared to the group only stimulated with SFN.

Stimulation with SFN (100 μg/mouse) *in vivo* could significantly promote the phosphorylation of ERK and p38 MAPK in the mouse peritoneal macrophages. Increased p-ERK1/2 and p-p38 MAPK, accompanying with decreased ERK and p38 MAPK were observed in macrophages after stimulation with SFN *in vivo* for 48 or 72 h (Fig. 4B). Different from *in vitro* assay, SFN could obviously increase both of JNK and p-JNK in macrophages *in vivo* (Fig. 4B). Thereby, SFN can activate p38 MAPK and JNK in macrophages *in vitro* and *in vivo*. However, ERK can not be activated by SFN *in vitro,* but can be done *in vivo*. This might be due to the different environments *in vitro* and *in vivo*.

### Surfactin activating NF-κB in macrophages

**Figure 5:**
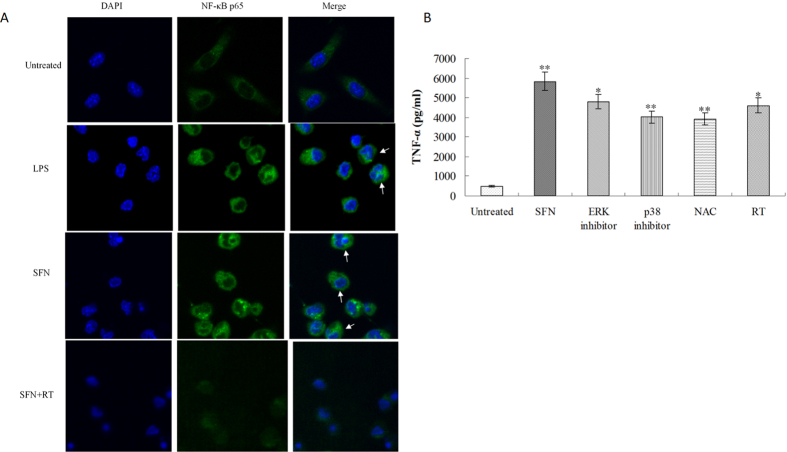
Surfactin activating NF-κB to produce TNF-α by macrophages. (**A**) Raw 264.7 cells were stained with FITC-labeled NF-κB p65 antibody and DAPI, then observed by Confocal microscopy (Magnification is 63X). NF-κB was only observed in the cytoplasm of spindle-shaped macrophages untreated by SFN, while both in the cytoplasm (green fluorescence) and nucleus (co-located with blue fluorescence) of star-shaped macrophages treated by SFN (directed by white arrows). The SFN-stimulated NF-κB nuclear translocation was obviously inhibited by the mitochondria-dependent ROS inhibitor of RT. (**B**) Surfactin promoting TNF-α production. SFN significantly promoted TNF-α production when compared to untreated control. Inhibition of ROS and inactivation of MAPKs could both significantly inhibit the SFN-stimulated TNF-α production by macrophages. The stars of SFN group indicate the significant difference between SFN and untreated control, and the star(s) of other groups indicate the significant difference from the SFN group.

Many adjuvants can evoke immune response through MAPKs signalling pathway to activate NF-κB that is mainly present in the cytoplasm of non-stimulated cells. Once upon to stimulation, NF-κB is activated and translocated into the nucleus^13^. After stimulation with 5 μg/ml SFN, Raw 264.7 cells were stained with FITC-labeled NF-κB p65 antibody and DAPI, then observed by Confocal microscopy. The results clearly showed that SFN could activate NF-κB nuclear translocation (Fig. 5A). In the non-stimulated control group, NF-κB was only observed in the cytoplasm of spindle-shaped macrophages. In response to SFN or LPS stimulation, NF-κB was observed both in the cytoplasm and the nucleus (stained by DAPI with blue fluorescence) of star-shaped macrophages, indicating that NF-κB was activated by SFN following translocated into the nucleus of activated macrophages. Further investigation found the SFN-stimulated NF-κB nuclear translocation was obviously inhibited by the mitochondria-dependent ROS inhibitor RT, suggesting that SFN activates NF-κB by the mitochondria-dependent ROS signalling pathway.

### Surfactin promoting TNF-α production in macrophages

As described above, SFN could activate MAPKs signalling pathway and NF-κB in macrophages. As a result, the pro-inflammatory cytokine of TNF-α was produced by macrophages after stimulation with SFN *in vitro* for 8 h (Fig. 5B). Both SB203580 (p38 MAPK inhibitor) and PD98059 (ERK inhibitor) could significantly inhibit the SFN-induced TNF-α production by macrophages. Similarly, ROS inhibitors (NAC or RT) could also inhibit the SFN-stimulated TNF-α production by macrophages when compared to the SFN group. The results indicate that SFN can promote macrophages to produce TNF-α for induction of immune response via the ROS/MAPKs-dependent signalling pathway.

### Surfactin inducing macrophages apoptosis

**Figure 6:**
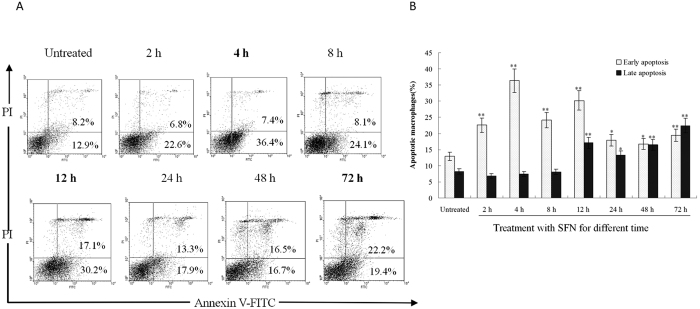
Surfactin inducing macrophages apoptosis. (**A**) Mice were stimulated with SFN (100 μg/mouse) *in vivo*, then the apoptosis of peritoneal macrophages was determined by staining with Annexin V-FITC and PI. (**B**) The percentage of Annexin V-FITC-stained cells (early apoptotic cells) was significantly higher than the untreated control at the time points of 4 h and 12 h. Post administration more than 12 h, the lately apoptotic cells (stained by Annexin V-FITC and PI) significantly increased when compared to untreated group. Double stars (p < 0.01) and single star (p < 0.05) mean the significant difference between SFN treatment and untreated.

It is well known that ROS can induce cell apoptosis^14^. In this study, ROS were induced in the mouse peritoneal macrophages after stimulation with SFN *in vivo*, then the apoptosis of macrophages was determined by staining with Annexin V-FITC and PI. The results showed the percentage of Annexin V-FITC-stained cells (early apoptotic cells) was significantly higher than the macrophages untreated with SFN at the time points of 4 h and 12 h after administration (Fig. 6). Post administration more than 12 h, the lately apoptotic cells (stained by both Annexin V-FITC and PI) significantly increased when compared to the untreated group (Fig. 6). The time points for apoptosis were consistent with the ROS robust in macrophages (Fig. 1B), indicating that SFN stimulates to produce ROS for induction of macrophages apoptosis *in vivo*. Macrophages can engulf apoptotic debris^15^, then DAMPs such as ATP, uric acid, DNA and RNA in apoptotic debris will activate inflammasomes in macrophages.

### Surfactin activating inflammasomes in macrophages

**Figure 7:**
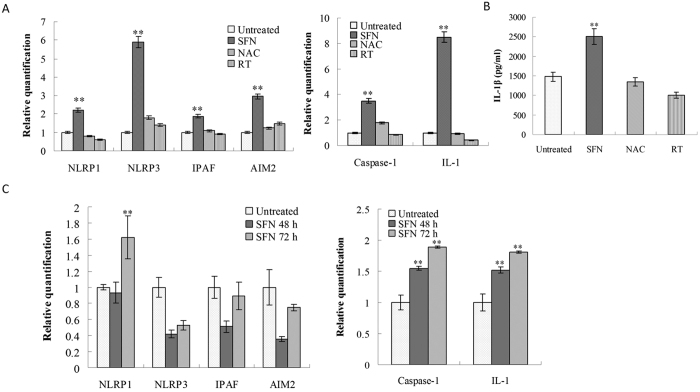
Surfactin stimulating inflammasomes in macrophages. (**A**) Stimulation with SFN *in vitro*. SFN (5 μg/ml) obviously increased genes transcription of four inflammasomes (NLRP1, NLRP3, IPAF and AIM2), caspase-1 and IL-1 in Raw 264.7 cells, which could be inhibited by pre-treatment with ROS inhibitors (NAC or RT). (**B**) SFN significantly promoted IL-1β production by Raw 264.7 cells, which could be inhibited by pre-treatment with ROS inhibitors. (**C**) Stimulation with SFN (100 μg/mouse) *in vivo*. SFN significantly promoted NLRP1, caspase-1 and IL-1 transcription in the peritoneal macrophages. The group with stars is significantly different from other groups.

SFN penetrates into macrophages binding to mitochondria, promoting ROS production, and inducing cells apoptosis to release DAMPs. Thereby, SFN is potential for activating inflammasomes by itself (as a ligand), ROS or DAMPs in macrophages. After analysis by RT-PCR, it was found SFN (5 μg/ml) could significantly increase four inflammasomes (NLRP1, NLRP3, IPAF and AIM2), caspase-1 and IL-1 in Raw 264.7 cells. Pre-treatment with NAC or RT (ROS inhibitors) could significantly decrease SFN-stimulated inflammasomes, caspase-1 and IL-1 in macrophages (Fig. 7A). IL-1β produced by Raw 264.7 was determined by ELISA. The results showed SFN (5 μg/ml) could significantly promote IL-1β production by Raw 264.7 when compared with the untreated control. Pre-treatment with NAC or RT could significantly inhibit SFN-stimulated IL-1β production by macrophages (Fig. 7B). The results indicate that SFN can activate inflammasomes to produce IL-1β for induction of immune response.

As shown in Fig. 7C, *in vivo* stimulaion with SFN (100 μg/mouse) could increase both NPLR1, caspase 1 and IL-1 transcription in macrophages when compared to the untreated control. Instead, IPAF and AIM2 were both less than the control post-stimulation for 48 h, and similar to the control post-stimulation for 72 h. Surprisingly, *in vivo* stimulation with SFN led to a decrease of NLRP3 in macrophages both at 48 and 72 h, which was different from *in vitro* assay. The results show that SFN can activate macrophages to form NLRP1 for promotion of IL-1 production *in vivo*.

## Discussion

Adjuvants are very important for developing effective vaccines. In addition to the widespreadly used alum adjuvant, other types of adjuvants have also been studied in recent years. Particularly, PAMPs (pathogen-associated molecular patterns) are well known for their ability to activate innate immunity after being recognized by PRRs (pattern recognition receptors). The secondary metabolitic lipopeptide of surfactin (SFN) is able to act both as parenteral and non-parenteral adjuvant for enhancing immunity in mice^7^,^8^. SFN can penetrate into antigen present cells to induce ROS, enhance cytokines production and promote costimulatory molecules expression^7^,^8^. However, little is known about its mechanisms of action.

SFN has been reported with a popular activity to induce ROS in plant, fungal and animal cells^7^,^9^,^10^. In this study, SFN induced ROS via mitochondria in macrophages. After incubation, SFN penetrated into macrophages binding to mitochondria. This binding was clearly observed under Confocal microscopy (Fig. 3). SFN can also bind to ATPase on mitochondria, following induction of ROS in fungal cells^10^. The structure of SFN comprises of a heptapeptide (ELLVDLL) linked to a β-hydroxy fatty acid chain via a cyclic ester^16^,^17^. Presence of two acidic amino acids (Glu and Asp) in the heptapeptide group forms a “claw” like structure, which is a potential binding site for cations^16^,^17^,^18^. Otherwise, the pI value of SFN is about 2.0 due to the presence of these two acidic amino acid residues, and the molecule will hold negative charges in a neutral pH solution. The negative charges are necessary for the “detergent-like” role of SFN on membranes. SFN firstly inserts into cell membrane by hydrophobic interactions, then the electrostatic interactions between SFN and cell membrane (with negative charges) will act as a “crowba” to disturb cell membrane^17^,^19^. In this study, SFN could penetrate into the cytoplasm of macrophages by hydrophobic and electrostatic interactions between SFN and cell membrane. It is well known the proton is pumped out for maintaining a proton gradient across the mitochondrial membrane for production of ATP in cells. After penetration, SFN with negative charges might bind to proton to destroy the proton gradient across the mitochondrial membrane. As a result, the electron transfer process was arrested following with ROS production in macrophages by a pathway dependent on mitochondria.

There are several pathways to produce ROS in cells, including mitochondria, NADPH, Xanthine oxidase, cyclooxygenase, *etc*.^20^. Here, the mitochondria-dependent ROS inhibitor (Rotenone plus TTFA) could obviously inhibit ROS production in macrophages, suggesting that SFN induces ROS via mitochondria. SFN also induced macrophages to produce ROS *in vivo*. After *i.p.* injection with SFN, ROS were induced by a mode of repeated increase and wane in the mouse peritoneal macrophages, different from the results *in vitro*. This is explained that SFN can consistently induce ROS in macrophages, and the accumulated ROS activate the ROS clearance systems as a feedback *in vivo*.

ROS are reported with multiple functions including activation of mitogen activated protein kinases (MAPKs). MAPKs family includes extracellular signal-regulated kinase (ERK), p38 mitogen-activated protein kinase (p38 MAPK) and Jun N-terminal kinase (c-JNK)^11^,^12^. In this study, treatment with SFN could increase p38 MAPK, JNK, p-p38 MAPK and p-JNK in macrophages, and inhibition of ROS only decreased p38 MAPK and JNK expression, but not their phosphorylation (Fig. 4). This result indicates the SFN-induced ROS only contribute to increase p38 MAPK and JNK expression but not their phosphorylation in macrophages. SFN may promote MAPKs phosphorylation by other mechanisms. Otherwise, ERK was activated by SFN *in vitro* but not *in vivo*. There are several reasons for the difference between *in vitro* and *in vivo* for activation of ERK signalling pathway. For example, different macrophages were used in this study. Raw264.7 was used for *in vitro* assay but the peritoneal macrophages were used for *in vivo* assay. Also the treatment time was different between *in vitro* and *in vivo*, it was 8 h for *in vitro* assay while 72 h for *in vivo* assay.

Many adjuvants can evoke immune response through MAPKs signalling pathway to activate NF-κB. For example, TLR agonists such as CpG DNA motifs can activate MAPKs and NF-κB, following lead to secretion of inflammatory mediators, expression of costimulatory ligands and major histocompatibility complex (MHC) molecules, ultimately enhance host humoral and cellular immune response^3^,^5^,^21^. In this study, SFN activated NF-κB nuclear translocation that was obviously inhibited by the mitochondria-dependent ROS inhibitor. The results suggest the mitochondria-dependent ROS signalling pathway is necessary for activation of NF-κB by SFN. Controversially, Park reported that SFN could inhibit the activation of NF-κB in macrophages^22^. The results are from the different context of experiments. We directly stimulated macrophages with SFN, while Park firstly stimulated macrophages with LPS following with SFN^22^.

TNF-α production was stimulated by SFN, but partially inhibited by kinase inhibitors of p38 and ERK, and ROS inhibitors of NAC and RT. This result indicates that ROS/MAPKs is not the only pathway in regulating TNF-α production under SFN stimulation. Other signaling pathways are possible to regulate TNF-α production *in vitro* and *in vivo*. For example, TLRs can activate NF-κB to produce TNF-α including TLR2, TLR4, TLR5, TLR7 and TLR9^23^. JAK/STAT signaling pathway is also reported to regulate TNF-α expression^24^. Otherwise, the initial TNF-α can act as an inducer to activate NF-κB to produce TNF-α and other cytokines by the RIP1-MEKK3-TAK1-IK signaling pathway^25^.

ROS are reported with multiple functions. In addition to activation of MAPKs and NF-κB, ROS also induce cells apoptosis *in vitro* and *in vivo*^14^,^15^. Here, treatment with SFN could significantly increase macrophages apoptosis. In the mouse peritoneal cavity, SFN promoted macrophages apoptosis via induction of ROS. Similarly, other adjuvants like cationic liposome adjuvant, could also induce ROS to promote cell apoptosis, which played a central role for the activity of liposome adjuvanted cancer vaccine^26^. These events are associated with endogenous danger signals (DAMPs), particularly, uric acid and host DNA are released from the apoptotic cells following trigger alarm and inflammation^27^.

In this study, DAMPs released from the SFN-induced apoptotic cells could activate 4 inflammasomes (NLRP1, NLRP3, IPAF and AIM2)^28^, caspase-1 and IL-1 in macrophages; however, this activation by SFN was significantly inhibited by pre-treatment with ROS inhibitors. These evidences verify that SFN can activate ROS-dependent inflammasomes to promote IL-1 release and induce immune response, and therefore ROS may represent an obvious link between SFN and its adjuvanticity. However, the *in vivo* results were different from *in vitro*. For example, NLPR1, NLPR3, IPAF and AIM2 were all significantly activated by SFN *in vitro*, but only NLRP1 was activated *in vivo*. This may be explained by different macrophages used for *in vitro* (Raw 264.7) and *in vivo* (peritoneal macrophages), and different SFN-treatment time between *in vitro* (8 h) and *in vivo* (48 h and 72 h, respectively). In addition to SFN, several other adjuvants have also been reported for activation of inflammasomes to trigger immune response such as alum^29^, the AS04 adjuvant system^30^, the mycobacterial cord factor trehalose-6, 6-dimycolate^31^, *etc*.

We previously showed that intragastric treatment of SFN significantly reduced TNF-α level in the atherosclerotic lesions of ApoE^−/−^ mouse^32^, which was different from stimulation of macrophages to produce TNF-α in this study. By *i.g.* treatment, SFN mainly stimulated the innate immune system in the intestine by induction of regulatory T cells and cytokines bias to Th2 type humoral immunity. Possibly, as a kind of non-pathogen-associated molecular patterns of *Bacillus*, SFN might talk the intestinal mucosal immune system that the mucosal immune response should be elicited to produce IgA for limitation of *Bacillus* out of the mucosa by induction of anti-inflmmatory cytokines such as IL-4, IL-10 and TGF-β. Indeed, it is not clear whether SFN could directly regulate macrophages in the atherosclerotic lesions, and possibly it plays the anti-atherosclerotic role by induction of anti-inflammator cytokines such as IL-10 and TGF-β for inhibition of Th1-type immune response in the atherosclerotic lesions. However, once penetrating through the intestinal mucosa into blood, the immune system will react fast to bacteria by induction of inflmmatory cytokines such as TNF-α. Thereby, with intraperitoneal injection, SFN can directly interact with peritoneal macrophages, and stimulate these cells to produce TNF-α eliciting inflammation *in vivo*.

**Figure 8:**
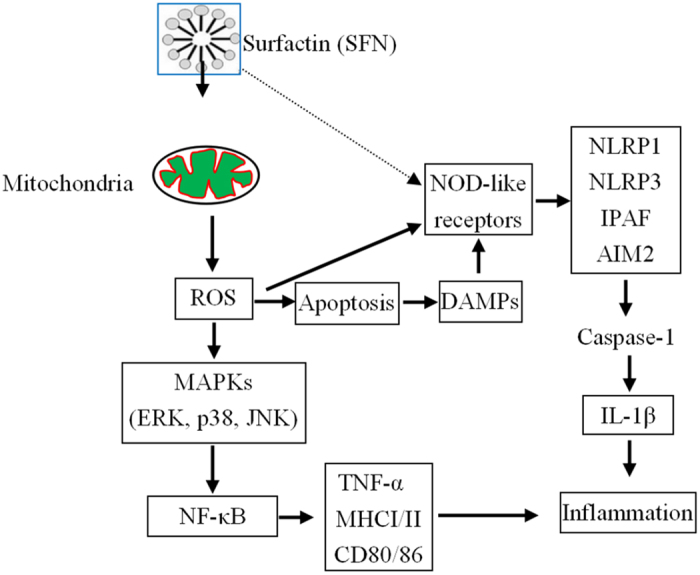
Overview of signalling pathways stimulated by surfactin in macrophages (for details see main text). The solid and dashed arrows illustrate the verified and possible pathways of surfactin in macrophages, respectively.

In this study, SFN modulates host innate immunity by multiple signalling pathways, including ROS, MAPKs, NF-κB and inflammasomes (Fig. 8). The SFN adjuvant activates innate immunity, induces specific genes transcription, and modulates NF-κB activity via MAPKs by the mechanisms that appear to be distinct from both the typical toll-like receptors agonists and other adjuvants like alum or MF59^33^,^34^. Firstly, SFN induces ROS in macrophages, indicating SFN can evoke immune response by the ROS-dependent signalling pathway. Secondly, SFN penetrates into cells then bind to mitochondria, suggests it can activate immune response by the mitochondria-dependent pathway. Thirdly, SFN is possible to act as a kind of non-pathogen-associated molecular patterns for some intracellular NOD like receptors to activate inflammasomes. Lastly, SFN can also induce cells apoptosis to release DAMPs for activating inflammasomes in macrophages.

## Materials and Methods

### Preparation of surfactin

Surfactin (SFN) was purified from *Bacillus amyloliquefaciens* WH1 culture and then characterized as methods described previously^10^. Endotoxin content in SFN was less than 0.05 EU/μl after being detected with LAL kits (Cape Cod, USA) by the directions of manual.

### Detection of ROS in surfactin-stimulated macrophages

For *in vitro* assay, RAW 264.7 cells (1 × 10^6^/ml) were incubated in 96-well plates with DMEM medium for 24 h. Then SFN was added to each well at a final concentration of 2.5, 5.0 and 7.5 μg/ml, respectively. Lipopolysaccharide (LPS, Sigma-Aldrich, USA) was added to the control wells at a final concentration of 1 μg/ml. Negative control was set up by addition with medium. The plates were further incubated at 37 °C for 1, 2, 4, 8 and 12 h in a CO_2_ incubator, respectively.

Balb/C mice (male, 5–6 weeks old, n = 6 per group) were obtained from the Center for Disease Prevention and Control of Hubei province, China, and housed under standard pathogen-free conditions following the University Ethics Committee’s guidelines. The experiments were approved by the Ethical Committee for Animal Experiments of the Huazhong Agricultural University, and performed in accordance with their guidelines. For *in vivo* assay, the mice were *i.p.* injected with SFN at a dose of 100 μg/mouse or just normal saline. After administration for 1, 2, 4, 8, 12, 24, 48 and 72 h, macrophages were collected from the peritoneal cavity of mice for the following studies.

The SFN-stimulated macrophages *in vitro* and *in vivo*, were loaded with DCFH-DA, a fluorescent probe for ROS (Sigma-Aldrich, USA) at 10 μmol/l in all wells. After further culture for 20 min in dark, the cells were collected and detected by FACS after fixation by 4% (w/v) paraformaldehyde solution^7^.

To investigate the pathway of SFN to induce ROS in macrophages, Raw264.7 cells were cultured directly on glasscover-slips in 35 mm diameter dishes with different ROS inhibitors (Table 1, Sigma-Aldrich, USA)^20^, then stimulated with SFN at 5 μg/ml for 8 h. Cells without stimulation by SFN, or just stimulation with SFN (5 μg/ml) or LPS (1 μg/ml) but without ROS inhibitors were used as controls. After incubation, cells were loaded with DCFH-DA at 10 μmol/l in dark for 10 min. Thereafter, cells were washed twice by 1 ml PBS, then observed by Confocal microscopy (LSM 510 Meta microscope, Zeiss, Jena, Germany) with an excitation wavelength of 488 nm and an emission wavelength of 525 nm after fixation by 4% (w/v) paraformaldehyde solution.

### Determining intracellular localization of surfactin in macrophages

SFN was labeled with NHS-Rhodamine B (Thermo, USA) in our lab by the directions of manual. Raw 264.7 cells (5 × 10^5^ cells/ml) were cultured directly on glasscover-slips in 35 mm diameter dishes with NHS-Rhodamine B-labeled SFN at 5 μg/ml for 30 min in dark, then added with Mito-Tracker Green (fluorescent probe for staining mitochondria, Sigma-Aldrich, USA) at 100 nmol/l. Thereafter, the cells were incubated at 37 °C for 30 min in dark, then stained with DAPI at 1 μg/ml for 10 min, following observed by Confocal microscopy after fixation by 4% (w/v) paraformaldehyde. For confocal microscopy, the excitation and emission wavelength was 555 nm and 580 nm for NHS-Rhodamine B, and 490 nm and 516 nm for Mito-Tracker Green, respectively.

### Analysis of MAPKs in surfactin-stimulated macrophages by Western blot

Raw 264.7 cells were adjusted to 2 × 10^6^/ml, cultured with or without Rotenone plus Thallium trifluoroacetate (10 nmol/ml, Mitochondria-dependent ROS inhibitor, Table 1) for 1 h, then stimulated with SFN (5 μg/ml) for 8 h. The cells added with medium were used as control (untreated). After stimulation, cells were harvested and lysed in ice-cold lysis buffer consisting of 1% Triton X-100, 1% deoxycholate, and 0.1% sodium dodecyl sulfate for 30 min. The supernatants were collected by centrifugation at 8,000 rpm, then stored at − 70 °C.

Balb/C mice (n = 6 per group) were stimulated by *i.p.* injection with 100 μg/mouse SFN for 48 and 72 h, respectively. The mice injected with normal saline were used as control. After stimulation, macrophages were collected from the peritoneal cavity by a 2-ml syringe and adjusted to 2 × 10^6^/ml, then lysed for collecting supernatants as described above.

The protein quantity of cell lysates were determined using Bradford reagent (Bio-Rad; Hercules, CA, USA). Then, the protein samples (50 μg total proteins) were separated by 12% SDS-polyacrylamide gel electrophoresis, transferred to a polyvinylidene difluoride membrane, and exposed to the appropriate antibodies including ERK (Biolegend, USA), p-ERK1/2 (Biolegend, USA), p38 MAPK (Biolegend, USA), p-p38 MAPK (Santa Cruz, USA), JNK (Biolegend, USA), p-JNK (Biolegend, USA) and β-Actin (Santa Cruz, USA). The proteins on membrane were visualized by the enhanced chemiluminescence detection system (Amersham Biosciences, Piscataway, NJ, USA) using horseradish peroxidase-conjugated anti-rabbit or anti-mouse secondary antibodies. Images were acquired using an ImageQuant 350 analyzer (Amersham Biosciences).

### Determination of NF-κB in surfactin-stimulated macrophages

Raw 264.7 cells were adjusted to 5 × 10^5^/ml, cultured directly on glasscover-slips with or without the mitochondria-dependent ROS inhibitor of Rotenone plus Thallium trifluoroacetate (RT, Table 1) for 1 h, then stimulated with SFN at 5 μg/ml for 8 h. The cells added with medium were used as control (untreated). After stimulation, the cells were fixed by 1 ml 4% paraformaldehyde at room temperature for 15 min, permeabilized with 100% methanol for 10 min, then stained with a 1:20 dilution of FITC-labeled NF-κB p65 monoclonal antibody (Santa Cruz, USA) at 10 μg/ml and DAPI at 1 μg/ml for 1.5 h, following observed by Confocal microscopy. For confocal microscopy, the excitation and emission wavelength was 488 nm and 519 nm for FITC, and 358 nm and 461 nm for DAPI, respectively.

### Detection of TNF-α and IL-1β produced by surfactin-stimulated macrophages

Raw 264.7 cells were adjusted to 1 × 10^6^/ml, cultured in 96-well plates for 200 μl per well with ROS inhibitors (NAC or Rotenone plus Thallium trifluoroacetate, Table 1), or MAPKs inhibitors (PD98059 or SB203580, Table 2) for 1 h, then stimulated with SFN (5 μg/ml) for 8 h and 12 h, respectively. Otherwise, some wells were only stimulated with SFN (5 μg/ml) without ROS inhibitors and MAPKs inhibitors. The cells only added with medium were used as negative control (untreated). After incubation, the supernatants were collected for determining TNF-α and IL-1β by ELISA kits (eBioscience, USA).

### Determining macrophage apoptosis induced by surfactin

Balb/C mice (male, 5–6 weeks old, n = 6 per group) were stimulated with 100 μg/mouse SFN for 0, 2, 4, 8, 12, 24, 48 and 72 h, respectively. At different time points, macrophages were collected from the peritoneal cavity of mice by a 2-ml syringe, then determined for possible apoptosis with commercial Kits by the directions of manual (Beyotime Biotechnology Co. Ltd, China).

### Assaying inflammasome genes transcription by RT-PCR

Raw 264.7 cells were adjusted to 2 × 10^6^/ml, cultured in 6-well plates at 1 ml per well with ROS inhibitors (NAC or Rotenone plus Thallium trifluoroacetate, Table 1) for 1 h, then stimulated with SFN (5 μg/ml) for 8 and 12 h, respectively. Otherwise, some wells were only stimulated with SFN (5 μg/ml) without ROS inhibitors. The cells only added with medium were used as negative control (untreated). After incubation, the cells (1 × 10^6^) were used for extracting RNA by Spin Mini RNA isolation kits (GE Healthcare, Buckinghamshire, UK) according to the manufacturer’s instructions.

Balb/C mice were *i.p.* injected with 100 μg/mouse surfactin and stimulated for 48 and 72 h, respectively. The mice only *i.p.* injected with normal saline solution were used as control. After stimulation, macrophages were collected from the peritoneal cavity by a 2-ml syringe, then 1 × 10^6^ cells were used for extracting RNA.

One microgram of total RNA was reverse-transcribed using Maxime RT PreMix (Promega, USA) and anchored oligo-dT_15_-primers. Real-time PCR (RT-PCR) was performed with the SYBR Green Master Mix (Applied Biosystems, Foster City, CA, USA) in a Chromo4 instrument (Bio-Rad). The relative amount of target mRNA was determined using the Ct method by normalizing target mRNA Ct values to those for β-actin (Ct). RT-PCR cycling conditions were 95 °C for 6 min; 45 cycles for 30 s at 95 °C, 30 s at 60 °C, and 30 s at 72 °C; and fluorescence measurement. The primer sequences were listed in Table 3.

### Statistical analysis

Data are expressed as mean ± SD. Each experiment was repeated at least three times. Statistical analysis was performed with SPSS, version 16.0 software to determine significant differences. We used either one- or two-way ANOVA followed by Dunn’s post hoc tests for analyses. Values of *P < 0.05 and **P < 0.01 were considered statistically significant.

## Additional Information

**How to cite this article**: Gan, P. *et al*. Surfactin inducing mitochondria-dependent ROS to activate MAPKs, NF-κB and inflammasomes in macrophages for adjuvant activity. *Sci. Rep.* **6**, 39303; doi: 10.1038/srep39303 (2016).

**Publisher’s note:** Springer Nature remains neutral with regard to jurisdictional claims in published maps and institutional affiliations.

## Figures and Tables

**Table 1:** ROS inhibitors.

ROS inhibitors	Functions	Final concentrations
N-acetylcysteine (NAC)	Universal ROS inhibitor	1 μmol/ml
Rotenone + Thallium trifluoroacetate (RT)	Mitochondria-dependent ROS inhibitor	10 nmol/ml + 10 nmol/ml
Apocynin	NADPH-dependent ROS inhibitor	1 μmol/ml
Allopurinol	Xanthine oxidase-dependent ROS inhibitor	10 nmol/mL
Mefenamic acid	Cyclooxygenase-dependent ROS inhibitor	20 nmol/mL

**Table 2:** Kinase inhibitors of MAPKs.

ROS inhibitors	Functions	Final concentrations
PD98059	ERK inhibitor	20 nmol/mL
SB203580	p38 MAPK inhibitor	20 nmol/mL

**Table 3:** Primers for RT-PCR.

Genes	Sequences	Tm (°C)
*β-Actin*	F: 5′-AGAGGGAAATCGTGCGTGAC-3′R:	62.0
	5′-CAATAGTGATGACCTGGCCGT-3′	60.3
*NLRP1*	F: 5′-ACTGAGAATGATTCCGGCTGTC-3′	60.3
	R: 5′-CTTCATCATCAATGGCCTTTCG-3′	58.3
*NLRP3*	F: 5′-CTTCCTCATGGATGGCTTTG-3′	60.0
	R: 5′-GGGCAGCAGTTTCTTTCGGA-3′	62.0
*AIM2*	F: 5′-AGTACCGGGAAATGCTGTTG-3′	60.0
	R: 5′-CAGGTGGTCAGCTAACTCTG-3′	62.0
*IPAF*	F: 5′-CAACTCAGAGAACATCCCTGAC-3′	60.3
	R: 5′-CTGCCTTGTCCTGTGACTCTG-3′	62.3
*Caspase-1*	F: 5′-CCTTCATCCTCAGAAACAAAGG-3′	58.3
	R: 5′-CATTATTGGATAAATCTCTGAAGG-3′	55.3
*IL-1*	F: 5′-GATCCACACTCTCCAGCTGCA-3′	62.3
	R: 5′-CAACCAACAAGTGATATTCTCCATG-3′	59.3
